# Biomarkers and smart intracranial devices for the diagnosis, treatment, and monitoring of high-grade gliomas: a review of the literature and future prospects

**DOI:** 10.1093/noajnl/vdz013

**Published:** 2019-07-08

**Authors:** Umbreen Hafeez, Lawrence M Cher

**Affiliations:** 1 Olivia Newton-John Cancer Research Institute, Austin Hospital, Melbourne, Australia; 2 Latrobe University School of Cancer Medicine, Melbourne, Australia; 3 Department of Medical Oncology, Austin Hospital, Melbourne, Australia

**Keywords:** Biomarkers, high-grade gliomas, intracranial devices, intracranial implants

## Abstract

Glioblastoma (GBM) is the most common primary brain neoplasm with median overall survival (OS) around 15 months. There is a dearth of effective monitoring strategies for patients with high-grade gliomas. Relying on magnetic resonance images of brain has its challenges, and repeated brain biopsies add significant morbidity. Hence, it is imperative to establish a less invasive way to diagnose, monitor, and guide management of patients with high-grade gliomas. Currently, multiple biomarkers are in various phases of development and include tissue, serum, cerebrospinal fluid (CSF), and imaging biomarkers. Here we review and summarize the potential biomarkers found in blood and CSF, including extracellular macromolecules, extracellular vesicles, circulating tumor cells, immune cells, endothelial cells, and endothelial progenitor cells. The ability to detect tumor-specific biomarkers in blood and CSF will potentially not only reduce the need for repeated brain biopsies but also provide valuable information about the heterogeneity of tumor, response to current treatment, and identify disease resistance. This review also details the status and potential scope of brain tumor-related cranial devices and implants including Ommaya reservoir, microelectromechanical systems-based depot device, Alzet mini-osmotic pump, Metronomic Biofeedback Pump (MBP), ipsum G1 implant, ultra-thin needle implant, and putative devices. An ideal smart cranial implant will overcome the blood-brain barrier, deliver various drugs, provide access to brain tissue, and potentially measure and monitor levels of various biomarkers.

**Key points**

- Combining blood and cerebrospinal fluid–based biomarkers will help in noninvasive diagnosis and monitoring of patients with high-grade gliomas.
- In the future, smart cranial devices will not only deliver drugs to the tumors but also provide valuable information.

Glioblastoma (GBM) is the most common primary brain neoplasm, accounting for 47% of such tumors. Despite treatment with aggressive surgical resection followed by concurrent chemotherapy and radiotherapy, median overall survival (OS) is disappointing and ranges from 14.6 to 16.7 months.^1–5^

Magnetic resonance imaging (MRI) has been the most useful means of monitoring these tumors providing macroscopic structural information but limited molecular biological information. Separating treatment effects from radiation necrosis, pseudoprogression, pseudoresponse, and the progressive tumor is often difficult.^6,7^ While numerous techniques can be helpful [eg, cerebral blood volume (CBV), diffusion-weighted imaging (DWI), and fluoro-ethyl-tyrosine positron emission tomography scan (FET-PET)], they all have limitations.^7–9^ This is particularly difficult when such effects are unexpected (such as pseudoprogression associated with the use of depatuxizumab–mafotidin) or in the setting of immunotherapy or intra-tumoral viral therapies which trigger significant inflammatory responses.^10,11^

Repeated brain biopsies add not only significant morbidity but also have inherent sampling errors due to the heterogeneous nature of tumor.^12,13^ Besides, brain tumors are often difficult to access, and scarcity of tissue may limit mutational analysis. Biomarkers in tissue, CSF, and blood offer the potential for early detection of tumor progression. Currently, GBM does not have a reliable biomarker in serum or CSF. Data are emerging for the use of extracellular macromolecules (free nucleic acids, metabolites, and proteins), extracellular vesicles, circulating tumor cells (CTCs), circulating immune cells, endothelial cells and endothelial progenitor cells for the diagnosis, monitoring, and detection of recurrence in patients with GBM. These biomarkers can potentially also offer information about the tumor’s molecular profile, prognosticate patient survival, and predict treatment responses.

Changes in CSF can sensitively reflect pathological processes in the central nervous system, but cytological analysis of cerebrospinal fluid (CSF) has low sensitivity.^14^ Nonetheless, CSF can be a valuable source for potential biomarkers. Obtaining CSF samples from patients is invasive and can lead to various adverse effects like headache, infection, bleeding, cerebral herniation, and back pain.^15^

Hence, there is a need for a device that can be implanted in patients, which potentially can make access to CSF easier without requiring repeated lumbar punctures. Here we discuss various intracranial devices which allow access to CSF for diagnostic and therapeutic purposes, deliver single or multiple drugs to CSF or directly in the tumor tissue, control rate and timing of drug delivery, provide feedback about the electrical activity of targeted neurons, and sense biomarkers. However, none of these devices have all these features combined in one unit. As such, there is a great need for a smart cranial device that may identify disease relapse and drug resistance well before clinical signs and symptoms become evident, providing a unique opportunity for earlier intervention, which may lead to improved outcomes.

## Potential Biomarkers

The worldwide incidence of brain tumors is 19 per 100,000 person-years; 12 per 100,000 person-years for benign tumors; and 7 per 100,000 person-years for malignant tumors.^16^ Gliomas are now classified by type (astrocytoma, oligodendroglioma, ependymoma), grade (I–IV), and more recently by molecular markers.^17,18^ The presence of 1p/19q-codeletion, O^6^-methylguanine methyltransferase (MGMT) methylation, and mutations in the enzyme IDH1/2 are prognostic biomarkers for high-grade gliomas.^19–21^ Heterozygous mutations affecting the Krebs cycle enzyme isocitrate dehydrogenase gene 1 or 2 (IDH1/2) are seen in both low-grade gliomas and secondary GBM, and are correlated with improved survival.^18,22^ These mutations are strongly associated with the accumulation of oncogenic metabolite 2-hydroxyglutarate (2HG), which is a valuable diagnostic and prognostic biomarker of IDH1/2 mutant glioma.^23^ The most common mutation is IDH1 R132H, and other rare mutations in IDH1 are R132C to R132G and R132S.^24^ Quantification of 2-HG in human gliomas with IDH1 and IDH2 mutations can be carried out non-invasively by magnetic resonance spectroscopy. However, these techniques are still in development.^25,26^ Mutations in alpha-thalassemia/mental retardation syndrome X-linked (ATRX) are a marker for astrocytic lineage in diffuse gliomas.^27^ Inactivation of the phosphatase and tensin homolog (PTEN) tumor suppressor gene on chromosome 10 leads to progression from low-grade to high-grade gliomas.^28^ EGFR amplification, over-expression, or presence of a mutation such as EGFRvIII is present in approximately 50% of GBMs.^29,30^ The presence of these mutations in GBM provides a target for biomarker development. Here we list currently investigated biomarkers isolated from serum or CSF in patients with high-grade gliomas (Table 1) and discuss their potential role in diagnosis, prognostication, identifying treatment response, and early detection of disease resistance or recurrence.

**Table 1.** Summary of Serum and CSF biomarkers’ sensitivity and specificity in patients with high-grade gliomas

Biomarker	Biofluid	Sensitivity (%)	Specificity (%)	Reference
**Extracellular macromolecules**				
Circulating tumor DNAs				
MGMT and PTEN methylation	Serum	55	100	Lavon et al^32^
1p/19q co-deletion	Serum	51	100	Lavon et al^32^
IDH1 mutation	Serum	60	100	Boisselier et al^34^
MGMT, RASSF1A, p15INK4B and p14ARF methylation	Serum	50	100	Majchrzak-Celińska et al^33^
EGFR, PTEN and IDH1 mutations	CSF	58	NR	De Mattos-Arruda et al^35^
1p/19q-codeletion, TERT, TP53, PTEN, IDH1, EGFR, and ATRX mutations	CSF	NR	NR	Miller et al^37^
H3F3A, HIST1H3B mutations	CSF	87.5	100	Huang et al^38^
H3F3A, TP53, ATRX, PDGFRA, FAT1, HIST1H3B, PPM1D, IDH1, NF1, PIK3CA, ACVR1 mutations	CSF	83.8	97.3	Pan et al^39^
**Circulating microRNAs**				
Elevated: miR-340, miR-576-5p and miR-626; decreased: let-7g-5p, miR-7-5p, and miR-320	Serum	NR	NR	Dong et al^41^
Elevated miR-185	Serum	NR	NR	Tang et al^42^
Elevated miR-210	Serum	NR	NR	Lai et al^43^
Decreased miR-205	Serum	NR	NR	Yue et al^44^
Elevated miR-106a-5p; decreased miR-182, and miR-145-5p	Serum	NR	NR	Zhao et al^45^
Elevated miR-222-3p, miR-20a-5p, miR-106a-5p; decreased miR-182 and miR-145-5p	Serum	NR	NR	Zhao et al^45^
Elevated miR-10b, miR-21, and miR-200	CSF	91	99	Teplyuk et al^46^
Elevated miR-223, miR-451, and miR-711	CSF	NR	NR	Drusco et al^47^
**Metabolites**				
Elevated cysteine, lysine and 2-oxoisocaproic acid	Serum	NR	NR	Moren et al^50^
Elevated 2-HG, histidine and tryptophan metabolites	CSF	NR	NR	Locasale et al^53^
**Proteins**				
Elevated GFAP	Serum	76	100	Jung et al^54^
Elevated GFAP	Serum	86	85	Kiviniemi et al^55^
Elevated GFAP	Serum	85	70	Tichy et al^56^
Elevated GFAP	Serum	43.7	95	Vietheer et al^57^
Elevated BMP2, HSP70 and decreased CXCL10	Serum	96	89	Elstner et al^16^
Elevated GFAP, IGFBP-2, and YKL-40	Serum	65	78	Perez-Larraya et al^59^
Elevated MBP	CSF	NR	NR	Nakagawa et al^60^
Elevated VEFF and basic-FGF	CSF	NR	NR	Peles et al^61^
Elevated VEGF	CSF	NR	NR	Sampath et al^62^
Elevated VEGF-B, basic-FGF, blood clotting factor VIII, β-2 microglobulin, osteonectin, and attractin	CSF	NR	NR	Khawaja et al^63^
Elevated IL-6	CSF	NR	NR	Shen et al^64^
**Extracellular vesicles**				
Elevated miR-320, miR-574-3p, RNU6-1	Serum	87	86	Manterola et al^67^
Signature of 7 miRNAs (miR-182-5p, miR-328-3p, miR-339-5p, miR-340-5p, miR- 485-3p, miR-486-5p, and miR-543)	Serum	91.7	100	Ebrahimkhani et al^68^
Detection of EGFRvIII	Serum	28	100	Skog et al^70^
Elevated angiogenin, FGF, IL-6, TIMP-1, TIMP-2, and VEGF	Serum	NR	NR	
Detection of EGFRvIII and IDH1 mutations	Serum	85	80	Shao et al^71^
Elevated miR-21	CSF	87	93	Akers et al^66^
Detection of EGFRvIII mutation	CSF	61	98	Figueroa et al^69^
Detection of IDH1 mutation	CSF	62.5	100	Chen et al^72^
**Circulating tumor cells (CTCs**)				
CTCs	Serum	72 pre-radiotherapy 8 post radiotherapy	100	MacArthur et al^73^
CTCs	Serum	39	100	Sullivan et al^74^
CTCs	Serum	20.6	96.6	Muller et al^75^
CTCs	Serum	53.8	NR	Krol et al^76^
**Circulating immune cells, endothelial cells, and endothelial progenitor cells**				
FoxP3^+^T regulatory cells	Serum	NR	NR	Thomas et al^79^
Foxp3^−^IL-10-expressing T regulatory cells	Serum	NR	NR	Li et al^80^
CEP	Serum	NR	NR	Bennett et al^81^
CEC	Serum	NR	NR	Bennett et al^81^

NR, not reported; CEP, circulating progenitor cells; CEC, circulating endothelial cell.

## Extracellular Macromolecules (Nucleic Acids, Proteins, and Metabolites)

Detection of extracellular macromolecules (free nucleic acids, metabolites, and proteins) in serum and CSF for high-grade gliomas have begun to gain traction in early phase studies. Circulating tumor DNA (ctDNA) are isolated from serum with next-generation sequencing or digital polymerase chain reaction (PCR) technique.^31^ ctDNA can be utilized to identify various mutations such as IDH1 mutation, 1p/19q-codeletion, MGMT methylation, and mutations in PTEN. When compared with the tissue gold standard, the sensitivity for 1p/19q-codeletion is 51% while MGMT methylation status ranges from 50% to 55% with a specificity of 100%.^32,33^ Boisselier et al detected IDH1 mutation in ctDNA extracted from the serum of patients with glioma with a sensitivity of 60% and specificity of 100%^34^ (Table 1). Due to the low sensitivity of these biomarkers in serum ctDNA, researchers are analyzing CSF ctDNA, and recently EGFR, PTEN, and IDH1 mutations are detected in ctDNA extracted from the CSF of GBM patients with a sensitivity of 58% compared with 0% for serum^35^ (Table 1). Pentsova et al. identified IDH1, TP53, ATRX, PTEN, and PIK3CA mutations from ctDNA derived from CSF of 6 out of 12 glioma patients (50%) utilizing next-generation sequencing. They were able to identify patterns of tumor evolution and temozolomide (TMZ)-associated mutations.^36^ Miller et al isolated tumor-derived DNA from CSF of 42 out of 85 adult patients with gliomas (49.4%) using next-generation sequencing and identified 1p/19q-codeletion, TERT, TP53, PTEN, IDH1, EGFR, and ATRX mutations. They also showed that shedding of tumor DNA into the CSF was significantly associated with tumor progression, tumor burden, the spread of tumor toward the ventricular system and shorter median OS.^37^ Huang et al identified Histone H3 mutations (H3F3A and HIST1H3B) in ctDNA derived from CSF of children with diffuse midline gliomas with a sensitivity of 87.5% and specificity of 100%.^38^ Similarly, Pan et al identified H3F3A, HIST1H3B, TP53, ATRX, PDGFRA, FAT1, PPM1D, IDH1, NF1, PIK3CA, and ACVR1 mutations in ctDNA isolated from CSF of patients with brain stem gliomas (Table 1). The 2-year survival of patients with H3F3A and HIST1H3B mutations was only 11.6%, when compared with the IDH1-mutant (75%) groups.^39^ As all major genetic mutations can be readily identified from CSF-derived ctDNA, this can help in diagnosis, or complement tissue diagnosis for patients with midline gliomas. This can identify the need for immediate versus delayed therapeutic interventions in these patients where surgical biopsy is usually challenging.

MicroRNAs (miRNAs) are small RNAs that regulate the expression of messenger RNAs and play a crucial role in gene regulation.^40^ Highly stable extracellular miRNAs can be extracted from blood and CSF of both healthy subjects and patients diagnosed with gliomas by quantitative reverse-transcriptase PCR (qRT-PCR) technique (Table 1). Dong et al detected 139 miRNAs in serum of patients with GBM. Among these miRNAs, miR-576-5p, miR-340, and miR-626 were significantly overexpressed, and miR-320, let-7g-5p, and miR-7-5P were significantly lower.^41^ Other authors have reported high levels of miR-185 and miR-210, and low levels of miR-205 in the serum of patients with gliomas.^42–44^ High levels of miR-210 and low levels of miR-205 are associated with poor patient outcome.^42,44^ Zhao et al described 2 miRNA panels to predict estimated 2-year OS and disease-free survival in patients with GBM. One panel consists of 3 serum miRNAs (miR-106a-5p, miR-182, and miR-145-5p) to predict OS and the second panel consists of 5 serum miRNAs (miR-222-3p, miR-182, miR-20a-5p, miR-106a-5p, and miR-145-5p) to predict disease-free survival. Poor OS was associated with raised miR-106a-5p and decreased miR-182 and miR-145-5p in the first panel, while poor disease-free survival was associated with raised miR-222-3p, miR-20a-5p, miR-106a-5p and decreased miR-182 and miR-145-5p in the second panel.^45^

Teplyuk et al detected a significantly high level of miR-10b and mi-R21 in CSF of patients with GBM and patients with brain metastasis from breast and lung cancer. Raised levels of the miR-200 family were observed in CSF of patients with brain metastasis but not with other neuropathological conditions including GBM.^46^ Drusco et al detected high levels of miR-223, miR-451, and miR711 and absence of miR-935 in CSF of patients with GBM.^47^ Thus, the level of miRNAs in CSF can be used to diagnose GBM and distinguish it from metastasis from other malignancies.

There are only a few published studies, indicating specific metabolites isolated from serum or CSF of patients with high-grade gliomas (Table 1). Cysteine is an essential amino acid, which is a precursor for glutathione synthesis. Glutathione synthesis plays a vital role in glioma cell survival.^48^ Higher levels of glutathione synthetase have been linked to poor progression-free survival (PFS) in GBM.^49^ Moren et al noted increased levels of cysteine in serum isolated from patients with GBM and raised levels of lysine and 2-oxoisocaproic acid in the serum of patients with oligodendrogliomas using gas chromatography-time of flight mass spectrometry (GC-TOFMS).^50^ Indoleamine 2,3-dioxygenase (IDO) is a tryptophan catabolic enzyme, which is upregulated in 90% of patients with GBM.^51^ GBM patients with strong IDO expression has significantly worse OS than patients with weak expression.^52^ Locasale et al isolated tryptophan metabolites from CSF of patients with GBM using hydrophilic interaction chromatography and showed raised levels of metabolites involved in tryptophan and histidine metabolism (indole, indoleacrylic acid, anthranilic acid, and histidine) in patients with recurrent GBM, when compared with newly diagnosed GBM patients.^53^ The study authors have also described the raised level of 2HG in CSF of GBM patients, indicating the presence of IDH1/2 mutations.^53^ Hence, these metabolite biomarkers can provide valuable information about the cellular energy state and can identify disease recurrence.

Investigators have been searching for protein biomarkers in serum and CSF of patients with GBM. Glial fibrillary acidic protein (GFAP), which is highly expressed in glial cells, is the most widely described protein identified from the serum of patients with GBM. Recent studies have identified raised GFAP level in serum of patients with GBM with varying sensitivity and specificity to diagnose high-grade gliomas^54–56^ (Table 1). Vietheer et al measured serum GFAP levels using an immunofluorescence assay and reported that although initially raised serum GFAP concentration does fall after surgery but later in the course of the disease GFAP levels are not predictive of tumor recurrence in patients with GBM.^57^ To improve diagnostic accuracy, researchers are looking at combined assays of various proteins.

Elstner et al described an enzyme-linked immunosorbent assay (ELISA)-based serum protein profile consisting of 3 proteins; bone morphogenic protein 2 (BMP2), heat shock 70-kDa protein (HSP70), and chemokine ligand 10 (CXCL10) that can diagnose GBM with a sensitivity of 96% and specificity of 89%.^58^ Another serum profile consisting of 3 proteins such as thrombospondin-1 (TSP1), HSP70, and insulin-like growth factor-binding protein 3 (IGFBP3) identified patients who lived more than 15 months after surgical resection of GBM.^58^ Perez-Larraya et al described an ELISA-based 2-step diagnostic procedure, including the 3 biomarkers; GFAP, insulin-like growth factor binding protein 2 (IGFBP-2), and chitinase-3-like protein 1 (YKL-40) that exhibited an area under the curve of 0.77 for differentiating patients with GBM from those with non-glial brain tumors.^59^ Nakagawa et al reported that, in patients with malignant gliomas, the level of protein biomarker myelin basic protein (MBP) in CSF changes in relation to tumor growth or regression and might change with treatment response.^60^ The majority of patients with malignant gliomas also show increased levels of vascular growth factor (VEGF), basic fibroblast growth factor (b-FGF), and interleukin IL-6 in CSF when compared with normal subjects^61–64^ (Table 1). These novel protein biomarkers could serve as an additional diagnostic tool for patients with inoperable brain lesions.

## Extracellular Vesicles

Extracellular vesicles (EVs) are cell-derived membranous structures which originate from the endosomal system or are shed from the cell membrane.^65^ EVs encompass exosomes, microvesicles, and retroviruses like particles and apoptotic bodies. They are rich in nucleic acids and have been detected in serum and CSF derived from GBM patients (Table 1). qRT-PCR shows that Evs derived from CSF have high levels of miR-21, with a sensitivity and specificity of 87% and 93%, respectively, for diagnosis of GBM.^66^ Similarly, EVs derived from serum of GBM patients have shown increased levels of miR-320, miR-574-3p, and RNU6-1 with a sensitivity and specificity of 87% and 86%, respectively, for diagnosis of GBM.^67^ Ebrahimkhani et al described a signature of 7 miRNAs (miR-182-5p, miR-328-3p, miR-339-5p, miR-340-5p, miR- 485-3p, miR-486-5p, and miR-543) derived from exosomes of GBM patients, which can diagnose GBM with a sensitivity of 91.7% and specificity of 100%.^68^ CSF or serum-derived EVs can also be used to detect EGFRvIII mutation in patients with GBM with varying sensitivity and specificity^69–71^ (Table 1). Shao et al identified IDH1 mutation in EVs from serum using a relatively new magnetic nanosensor technology, and Chen et al have identified IDH1 mutation in EVs from CSF of patients with gliomas using novel techniques such as BEAMing (beads, emulsion, amplification, magnetics) PCR and droplet digital PCR (ddPCR).^71,72^ Hence, EVs not only can offer noninvasive early indication of tumor progression or recurrence but can also aid in the detection of IDH1 and EGFRvIII mutations. However, further studies in a larger longitudinal cohort of patients are needed before their incorporation in clinical practice. Skog et al have demonstrated the presence of angiogenic proteins such as angiogenin, FGF, IL-6, TIMP-1, TIMP-2, and VEGF in EVs.^70^ It can be presumed that these proteins promote angiogenesis and aggressiveness of GBM.

## Circulating Tumor Cells

Rogue tumor cells that separate from the primary tumor or a metastatic deposit and enter in blood circulation are called CTCs. So far CTCs have been identified in the serum of high-grade glioma patients, but no CTCs have been identified from CSF of patients with GBM. CTCs in epithelial malignancies are usually detected via cell surface expression of epithelial cell adhesion molecule (EpCAM) which is not present in GBM cells. MacArthur et al described a telomerase-based assay to detect CTCs in peripheral blood samples of patients (8 of 11, 72%) with high-grade gliomas.^73^ Sullivan et al identified CTCs from blood samples in 13 of 33 (39.3%) patients with GBM using a microfluidic device that removes leukocytes from blood samples, enriching for CTCs without requiring tumor cell-specific capture antibodies.^74^ Sullivan et al also demonstrated that the frequency of CTCs with EGFR amplification was similar to the frequency of patient-matched tumor cells with EGFR amplification. Muller et al identified CTCs in 29 of 141 (20.6%) of GBM patients using glial fibrillary acidic protein-directed antibodies.^75^ Krol et al isolated GBM CTCs from peripheral blood samples of 7 of 13 (53.8%) patients using immunostaining and exome-sequencing techniques, but they did neither find any association between the presence of CTCs and MRI volume nor any of those patients developed extracranial metastasis.^76^

## Circulating Immune Cells, Endothelial Cells, and Endothelial Progenitor Cells

Immune system evasion is a hallmark of cancer, and tumor-associated immune cells have been reported in patients with GBM. It is known that FoxP3^+^T regulatory cells are not present in normal brain tissue. Heimberger et al showed that FoxP3^+^T regulatory cells are more common in astrocytomas than in oligodendrogliomas and, as tumors became more malignant, the number of FoxP3+ Tregs in them increases.^77^ However, the presence of FoxP3^+^T regulatory cells in patients with GBM does not correlate with OS.^77–79^ Li et al demonstrated increased numbers of CD4^+^ Foxp3^−^ IL-10-expressing Type 1 T regulatory cells using surface marker expression in peripheral blood samples from patients with GBM when compared with healthy controls.^80^ Circulating endothelial progenitor cells (CEPs) are released from bone marrow in response to angiogenic stimuli and can serve as an indicator for angiogenesis. Circulating endothelial cells (CEC) are mature endothelial cells, which are detached from blood vessels and enter the bloodstream. Bennett et al reported that preoperative CEP concentration correlates with tumor blood volume; they have also shown that CEC concentration in peripheral blood sample decreases after GBM patients undergo surgical resection. However, neither CEC nor CEP showed correlation with PFS or OS.^81^

## Smart Intracranial Devices

**Fig. 1:**
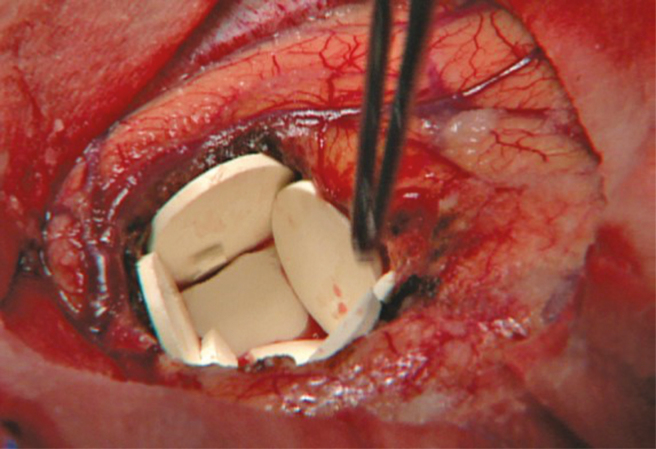
Carmustine wafers being implanted in the tumor resection cavity. (Photo courtesy of Henry Brem, MD, Johns Hopkins School of Medicine.)

**Fig. 2:**
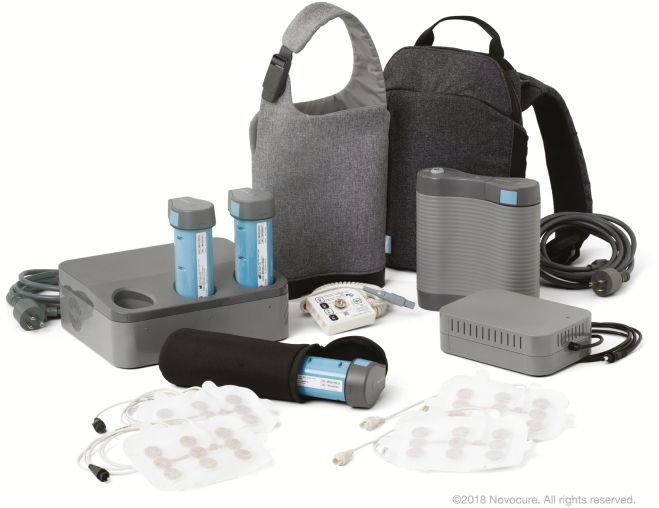
Optune^®^ previously known as Novo Tumor Treatment Fields^®^. (Photo courtesy of Novocure.)

The blood-brain barrier (BBB) not only acts as the rate-limiting factor in drug delivery to the brain; it can also limit the detection of potential biomarkers in serum. Approaches to circumvent the impermeability of the BBB and local tumor treatment are long being evaluated. They range from the direct introduction of chemotherapeutic agents by controlled release polymers placed in the resection cavity (Fig. 1), direct infusion of cytotoxic chemotherapy drugs in the tumor, and tumor treatment fields (Fig. 2).^82–84^ Other options include the use of nanocarriers and biocarriers. These carriers can enhance the permeability of therapeutic agents across the BBB, carry intracellular drugs, and release their payload into the brain parenchyma.^85^ Nanocarriers can take advantage of the enhanced permeation and retention effect that occurs in high-grade gliomas.^86,87^ Further clinical trials are awaited in this space.

**Fig. 3:**
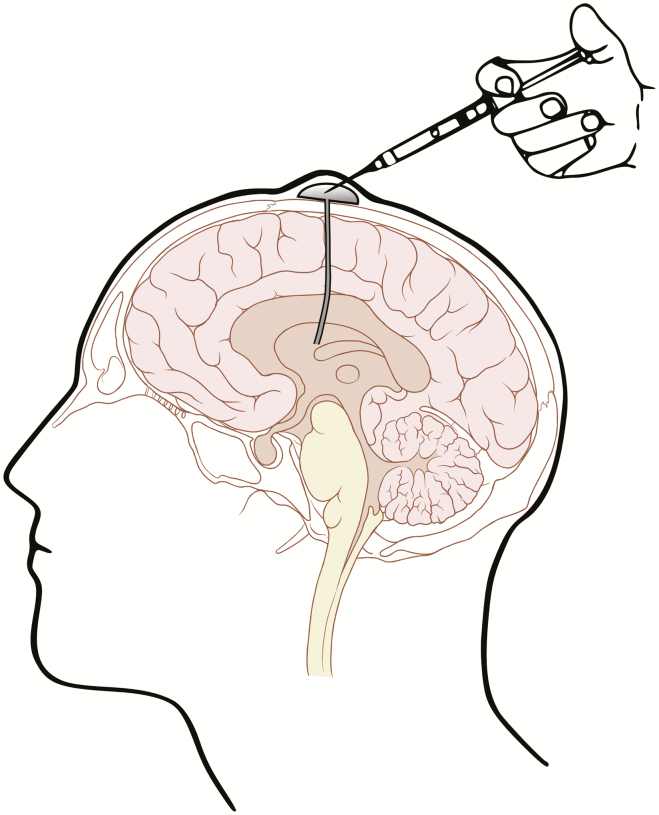
Ommaya reservoir.

To circumvent the BBB, there is a renewed interest in smart cranial devices that can deliver drugs directly to the brain tumors or the CSF. The Ommaya reservoir is an intraventricular catheter device which has been in use since 1963 (Fig. 3).^88^ The 3-cm reservoir is a mushroom-shaped, capsule with tubing going through a small hole in the skull into the lateral ventricle. It gives access to CSF without the need for repeated lumbar punctures. It is utilized for aspiration of CSF and administration of various drugs and chemotherapy most commonly for the leptomeningeal spread of carcinoma or leukemia.^89–91^ CSF aspirated through the device could possibly be used to monitor treatment response when an appropriate CSF biomarker has been identified. At times, the Ommaya reservoir is inserted into a progressive or recurrent tumor cyst to allow aspiration.^92^ Complications associated with the use of Ommaya reservoir are technical difficulties in placing reservoir, malfunction, misplacement, intraventricular hemorrhage, and intracranial infections.^93^ The risk of infection with this device is 5.5 to 8 % and, in most cases, it would require removal of the device.^94,95^

**Fig. 4:**
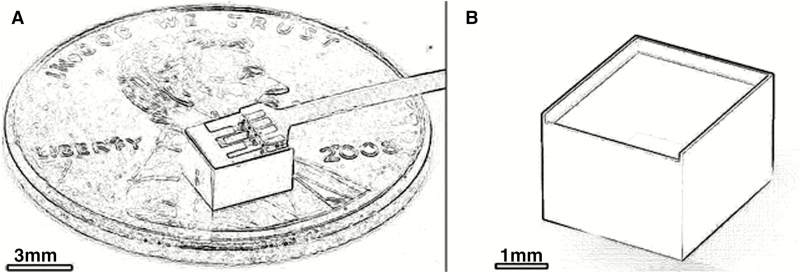
Photograph of the MEMS-based fully assembled device. The 3 green squares on the microchip are the suspended nitride membranes. The copper leads protrude from the device.

An intracranial microelectromechanical system (MEMS)-based depot device has been developed to deliver TMZ in a rodent glioma model (Fig. 4). This device is a liquid crystalline 1-mm polymer reservoir, capped by a microchip. The microchip contains 3 nitride membranes that can be independently controlled to release single or multiple drugs locally. Immunohistochemical studies showed that TMZ was released in a cytotoxic form. This device has shown promising results in a mouse model, with its ability to control the rate and timing of drug delivery via minute electric pulses. There were no complications of implanting this device in mice.^96^ The ability of this device to regulate drug delivery holds enormous potential for the management of intracranial tumors.

Bortezomib, a powerful cytotoxic drug, failed to show any activity in recurrent anaplastic glioma and GBM patients in phase II trials when combined with tamoxifen or vorinostat, due to poor penetration across BBB.^97,98^ However, the intracranial administration of bortezomib in glioma animal models has been shown to improve OS significantly compared with systemically administered drug. In this study, bortezomib was administered through Alzet mini-osmotic pumps.^99^ Alzet mini-osmotic pumps are small infusion pumps with a reservoir of 200 µL that deliver the drug at a predetermined rate. In this study, mini-osmotic pumps were implanted subcutaneously in scalp with a brain infusion kit to deliver bortezomib directly into the tumor tissue to circumvent the BBB. The study authors were unable to use imaging to determine tumor progression as pump placement interfered with imaging. Nonetheless, these pumps have the potential to deliver the drug where it is needed.

**Fig. 5:**
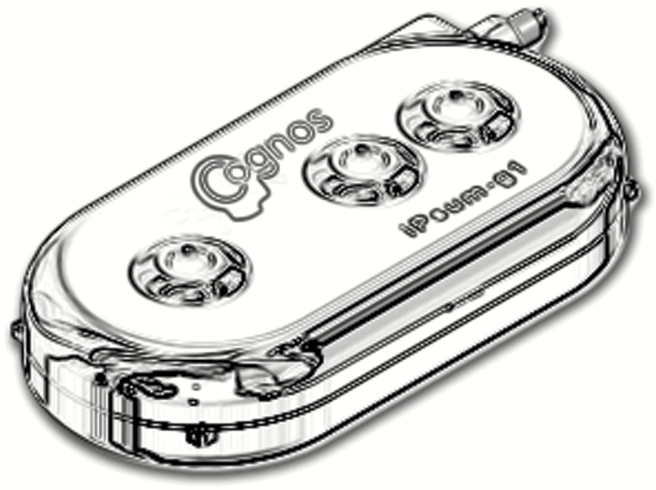
iPsum-g1, a smart implantable pump.

Pharmaco-Kinesis Corporation has developed an implantable Metronomic Biofeedback Pump (MBP), which is capable of delivering chemotherapy in a metronomic fashion with electronic feedback for patients with leptomeningeal carcinomatosis initially. It consists of a 2-lumen catheter; a microfluidic delivery pump with two 5-ml reservoirs and a spectrophotometer, which can provide real-time feedback to monitor chemotherapy concentrations in the CSF. It can be implanted in the chest, similar to a pacemaker, or the abdominal cavity. The pump is connected to an intracranial tumor or lateral ventricle by subcutaneous double lumen catheter. This device can be controlled via a remote wireless connection, sample CSF from the delivery site with an option to modify the treatment regimen. It has been tested in a swine model. Potential problems with the MBP device is poor drug circulation due to local tumor deposits, hydrocephalus, catheter occlusion, and communication malfunction. Phase I and II trials in humans are pending.^100^ Pharmaco-Kinesis Corporation also recently introduced iPsum-g1, a smart implantable pump, which can be implanted under the dura that delivers chemotherapy at scheduled intervals (Fig. 5). It has a biosensor, which can sense biomarker protein such as VEGF.^101^

**Fig. 6:**
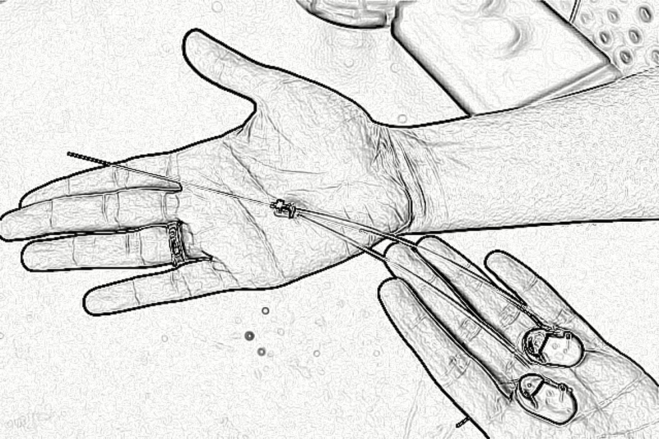
An ultrathin needle implant developed by Massachusetts Institute of Technology (MIT) scientists.

In January 2018, Massachusetts Institute of Technology scientists announced the development of an ultrathin needle implant that can deliver drugs directly to the brain (Fig. 6). The researchers made 2 ultra-thin medication tubes and slid them into a stainless steel needle that is about the diameter of a human hair. The needle is attached to 2 small, programmable pumps that can be implanted under the skin and contain the medications. An electrode on the tip can provide feedback about the electrical activity of targeted neurons after the medication is delivered. This system has been tested in mice animal models, but has not been used in humans yet.^102,103^

Bennett et al have developed boron-doped diamond-based synthetic electrodes that are capable of measuring neurochemicals (dopamine, norepinephrine, serotonin, and adenosine) released in human brain tissue.^104^ These electrodes are inserted in brain tissue during deep brain stimulation, a technique used to treat certain neuro-psychiatric conditions. On the basis of this technique, Prawer et al proposed to utilize diamond-coated flexible carbon fibers, to evaluate GBM in real time by measuring GBM-specific biomarkers like 2HG and relaying this information wirelessly. These diamond-coated flexible carbon fibers can also be utilized to deliver drugs of interest and measure drug levels of 5-(3-methyltriazen-1-yl) imidazole-4-carboxamide, the active metabolite of TMZ and monomethylauristatin F (MMAF), the anti-tubulin toxin used in the antibody–drug conjugate depatuxizumab mafodotin. Hence, this improved technique may allow real-time assessment of high-grade gliomas along with an enhanced pharmacokinetic assessment of new therapies.^105^ The limitations of this technique are that electrodes have to be inserted at the time of surgery.

## Summary

We now have a range of potential biomarkers that can be analyzed from serum or CSF of patients with gliomas, but their utility needs to be assessed in more detail, prospectively and longitudinally. Prospective serial measurements correlated with clinical and radiologic assessments are required to determine whether these biomarkers could accurately predict recurrence at an earlier stage and potentially differentiate between true progression versus pseudoprogression. The technologies required, however, are complex and varied. The sensitivity and specificity of these biomarker assays need to be improved to enter clinical practice. What remains to be understood is the clinical relevance of these biomarkers in patients with high-grade gliomas, as their presence in peripheral blood does not always seem to correlate with tumor aggressiveness or survival outcomes in this patient population.

Further improvements in technology, and combining blood and CSF-based analysis with imaging, will undoubtedly help in noninvasive diagnosis of patients with high-grade gliomas. This is paramount for those patients who are not optimal candidates for surgery due to underlying medical conditions or when surgical biopsy or resection is difficult due to the location of gliomas such as in patients with brain stem gliomas or when biopsy and imaging studies are inconclusive. Molecular profiling of high-grade gliomas may also help in monitoring treatment response and identifying disease resistance or recurrence. Identification of various mutations in serum or CSF will not only help in prognostication but will also direct clinicians toward targeted therapy. These biomarkers will also play a significant role in disease monitoring in pseudoprogression setting when imaging is not very helpful, and repeated brain biopsies are not desirable.

The intracranial devices offer the potential to permeate BBB and deliver drugs where they are needed, but they can be associated with various complications such as device misplacement, malfunction, intraventricular hemorrhage, intracranial infections, poor drug circulation due to local tumor deposits, hydrocephalus, catheter occlusion, and communication failure.^93,94,100^ In most of these devices, drugs are delivered through convection that can result in leakage of the convected drug in subarachnoid space and ventricles and cause transient chemical meningitis.^83^ Human studies of these devices had lagged behind, and few critical questions remain unanswered in terms of optimal location of these devices to deliver the drug to tumor cells not only within the tumor but also in adjacent parenchyma.^106^ Nevertheless, their placement requires neurosurgical expertise and meticulous care afterward, which can be a limiting factor in their widespread use. Further studies are warranted to create an ideal device that is capable of not only delivering drugs directly to the brain tumor, but it should also provide access to collect CSF or brain tissue samples. It should also be fitted with a microchip so that it can measure and monitor levels of various biomarkers and electric activity of neurons along with CSF pressure. Physicians should be able to get this information from the device in real time. It should be simple to implant, easy to operate, and maintain afterward. It should be able to be used throughout the treatment course of the patient.
